# Local Reports of Epidemic and Endemic Diseases

**Published:** 1855-12

**Authors:** 


### LOCAL REPORTS OF EPIDEMIC AND ENDEMIC DISEASES.

*During the Months of September, October, and November* 1855.

The subjoined Report is compiled from data furnished specially for this journal by medical men in twenty-two towns in England and Wales, viz.:

| Place. | County. | Lat. | Long. | Observer. |
|---|---|---|---|---|
| Odiham - - | Hampshire - | 51. 8 N. | 1. 3 W. | J. M'Intyre, M.D. |
| Bridgewater - | Somerset - | 51. 8 N. | 3. 0 W. | Alfred Haviland, Esq. |
| Canterbury - | Kent - - | 51.17 N. | 1. 4 E. | G. Rigden, Esq. |
| Chatham - | Kent - - | 51.24 N. | 0.14 E. | F. J. Brown, M.D. |
| Up. Holloway | Middlesex - | 51.32 N. | 0.03 W. | W. B. Kesteven, Esq. |
| Wanstead - | Essex - - | 51.32 N. | 0. 2 W. | F. Collins, M.D. |
| Swansea - | Glamorgansh. | 51.38 N. | 3.50 W. | W. H. Michael, Esq. |
| Saffron Walden | Essex - - | 52. 3 N. | 0.12 E. | T. Spurgin, Esq.<br>H. Stear, Esq. |
| Bedford - - | Bedfordshire - | 52. 8 N. | 1.51 W. | T. H. Barker, M.D. |
| Thetford - | Norfolk - - | 52.26 N. | 0.45 E. | H. W. Bailey, Esq. |
| East Dereham | Norfolk - - | 52.40 N. | 0.57 E. | J. Vincent, M.D. |
| Pontesbury - | Shropshire - | 52.43 N. | 2.50 W. | Wm. Eddowes, Esq. |
| Nottingham - | Nottinghamsh. | 52.50 N. | 1.10 W. | T. Robertson, M.D. |
| Wrexham - | Denbighshire - | 53. 2 N. | 3. 1 W. | E. Williams, M.D. |
| Hawarden - | Flintshire - | 53.11 N. | 3. 2 W. | T. Moffat, M.D. |
| Lincoln - | Lincolnshire - | 53.12 N. | 0. 5 W. | S. Lowe, Esq. |
| Alford - - | Lincolnshire - | 53.15 N. | 0. 6 E. | R. U. West, M.D. |
| Gainsborough | Lincolnshire - | 53.23 N. | 0.47 W. | D. Mackinder, M.D. |
| Liverpool - | Lancashire - | 53.24 N. | 2.59 W. | Thos. Bickerton, Esq. |
| Bolton - - | Lancashire - | 53.35 N. | 2.19 W. | W. H. Pendlebury, Esq. |
| Wst. Auckland | Durham - | 54.45 N. | 1.40 W. | G. Todd, Esq. |
| Rothbury - | Northumberld. | 55.25 N. | 1.50 W. | E. C. Summers, Esq. |

### QUARTERLY STATEMENT—No. III.

| Disease. | Where observed. | At what periods.—Weeks ending |
|---|---|---|
| 1. Scarlet fever - | Chatham - - | Oct. 26 |
| " | Upper Holloway | Oct. 5, 12 |
| " | Wanstead - | Sept, 7, 14; Oct. 5, 12 |
| " | Swansea - - | Every week but Sept. 7 [16, 23 |
| " | Saffron Walden | All Sept.; Oct. 12, 19, 26; Nov. 2, 9, |
| " | Bedford - - | Every week |
| " | Thetford - - | Sept. 7, 21, 28; Oct. 19, 26; all Nov. |
| " | East Dereham | Nov. 9, 16, 23, 30 |
| " | Pontesbury - | Oct. 5 |
| " | Nottingham - | All Oct.; Nov. 16, 23, 30 |
| " | Wrexham - | Oct. 12, 19, 26; Nov. 9, 16, 23, 30 |
| " | Hawarden - | Nov. 9 |
| " | Lincoln - - | Sept. 21, 28 |
| " | Alford - - | Nov. 9, 16, 23, 30 |
| " | Gainsborough - | Oct. 26; Nov. 2 |
| " | Liverpool - | Every week |
| " | Bolton-le-moors | Every week |
| " | West Auckland | All Oct. and Nov. |
| 2. Measles - - | Odiham - - | Sept. 7, 14, 21; Oct. 12 |
| " | Chatham - | All Sept. and Oct.; Nov. 23 |
| " | Upper Holloway | Oct. 5, 12, 26 |
| " | Saffron Walden | Oct. 19 |
| " | Thetford - - | Sept. 7, 14, 28; Oct. 5, 12, 26; all Nov. |
| " | East Dereham | Sept. 7 |
| " | Pontesbury - | Oct. 26; Nov. 2, 9 |
| " | Wrexham - | Sept. 28; Oct. 19; Nov. 16, 23, 30 |
| " | Alford - - | Oct. 26; Nov. 23, 30 |
| " | Liverpool - | Every week |
| " | Bolton-le-moors | Every week |
| 3. Small-pox - | Odiham - - | Nov. 2 |
| " | Canterbury - | Every week |
| " | Chatham - - | Oct. 26 |
| " | Swansea - - | Sept. 7, 28; Oct. 5 |
| " | Saffron Walden | Sept. 7 |
| " | Bedford - - | Sept. 14, 21, 28; Oct. 5 [2, 9, 16, 23 |
| " | Liverpool - | Sept. 7, 14, 28; Oct. 12, 19, 26; Nov. |
| " | Bolton-le-moors | Sept. 21, 28; Oct. 5, 12 |
| " | Odiham - - | Oct. 19 |
| 4. Hooping-cough | Canterbury - | Every week |
| " | Chatham - | Oct. 19; Nov. 16 |
| " | Upper Holloway | Oct. 5, 12 |
| " | Saffron Walden | Sept. 7; Oct. 12, 19; Nov. 16 |
| " | Bedford - - | Sept. 14; Oct. 12, 19 |
| " | Thetford - - | Sept. 21, 28; Oct. 5, 19, 26; all Nov. |
| " | East Dereham | Sept. 7; Oct. 26; all Nov. |
| " | Pontesbury - | Every week |
| " | Nottingham - | Sept. 21; Oct. 5, 26; Nov. 2, 9, 23, 30 |
| " | Lincoln - - | Every week but Nov. 30 |
| " | Alford - - | Oct. 26 |
| " | Gainsborough - | Every week [9, 16, 30 |
| " | Liverpool - | Sept. 21, 28; Oct. 5, 19, 26; Nov. 2, |
| " | Bolton-le-moors | Sept. 7, 14, 21; Oct. 12, 19, 26; Nov. |
| | | 2, 9, 16, 23 |
| " | Rothbury - | Oct. 12, 19 |
| 5. Croup - - | Bridgewater - | Nov. 9 |

| Disease. | Where observed. | At what periods.—Weeks ending |
|---|---|---|
| 5. Croup - - | Canterbury - | Nov. 9 |
| „ | Chatham - | Nov. 23 |
| „ | Swansea - - | Sept. 14; Oct. 5; Nov. 2 |
| „ | Saffron Walden | Oct. 5 |
| „ | Bedford - - | Sept. 14; Nov. 9 |
| „ | Nottingham - | Sept. 7, 14; Oct. 26; Nov. 30 |
| „ | Alford - - | Oct. 26; Nov. 16 |
| „ | Liverpool - | Oct. 19, 26; Nov. 23 |
| „ | Bolton-le-moors | Oct. 5, 12; Nov. 9 |
| 6. Catarrh - - | Odiham - - | Sept. 7, 14, 21; Oct. 19, 26; Nov. 2, 9, 16, 23 |
| „ | Bridgewater - | Nov. 23 |
| „ | Chatham - | Every week |
| „ | Wanstead - | Oct. 5 |
| „ | Swansea - - | Sept. 28; all Oct.; Nov. 2, 9, 30 |
| „ | Saffron Walden | Sept. 28; Nov. 9, 16, 23, 30 |
| „ | Bedford - - | All Nov. |
| „ | Thetford - - | Sept. 14, 21; Oct. 5; Nov. 2, 16, 23, 30 |
| „ | East Dereham | Sept. 28; Oct. 5, 12, 19 |
| „ | Pontesbury - | Nov. 9, 16, 23, 30 |
| „ | Nottingham - | Throughout the Quarter |
| „ | Hawarden - | Oct. 5, 19; Nov. 2, 30 |
| „ | Gainsborough - | Sept. 7, 14; all Oct. and Nov. |
| „ | Liverpool - | Sept. 14; Oct. 5, 12, 26; Nov. 9, 23 |
| „ | Bolton-le-moors | Sept. 21, 28; all Oct.; Nov. 2 |
| „ | Rothbury - | Nov. 23, 30 |
| 7. Influenza - | Odiham - - | Sept. 21; Nov. 9 |
| „ | Wanstead - | Nov. 30 |
| „ | Swansea - - | Sept. 7, 14, 21 |
| „ | Saffron Walden | Nov. 9, 16, 23, 30 |
| „ | Bedford - - | Oct. 26; all Nov. |
| „ | Thetford - - | Sept. 14, 21; Oct. 5, 12, 26; Nov, 2, 16, 23 |
| „ | Lincoln - - | Nov. 9, 16, 23 |
| „ | Nottingham - | Nearly every week |
| „ | Liverpool - | Sept. 28 |
| „ | Bolton-le-moors | Oct. 12, 19, 26; Nov. 2, 9, 16 |
| 8. Erysipelas - | Odiham - - | Sept. 7, 14, 21 |
| „ | Bridgewater - | Oct. 5 |
| „ | Canterbury - | Sept. 7, 28; Oct. 5, 12, 26; Nov. 2 |
| „ | Chatham - - | Oct. 5; Nov. 16, 23 |
| „ | Upper Holloway | Oct. 5 |
| „ | Wanstead - | Oct. 5, 26; Nov. 16 |
| „ | Swansea - - | Sept. 21 |
| „ | Saffron Walden | Oct. 19, 26; Nov. 2 |
| „ | Bedford - - | Nov. 2, 30 |
| „ | Thetford - - | Sept. 7, 21; Oct. 19; Nov. 9, 30 |
| „ | East Dereham | Nov. 23, 30 |
| „ | Pontesbury - | Sept. 21; Nov. 9, 16, 23 |
| „ | Nottingham - | Sept. 21, 28; Oct. 5, 12, 26; Nov. 23, 30 |
| „ | Wrexham - | Sept. 21; Oct. 19; Nov. 23, 30 |
| „ | Hawarden - | Oct. 19; Nov. 23 |
| „ | Alford - - | Sept. 14; Oct. 26; Nov. 2, 9 |
| „ | Gainsborough - | Sept. 7, 14 |
| „ | Liverpool - | Sept. 28; Oct. 5, 12; Nov. 9, 30 |
| „ | Bolton-le-moors | Sept. 14, 21, 28; Nov. 9, 16, 23, 30 |
| „ | West Auckland | Sept. 28 |
| „ | Rothbury - | Oct. 19, 26; Nov. 2 |
| 9. Cholera - - | Chatham - - | Sept. 7; Oct. 12; Nov. 2, 16 |

| Disease. | Where observed. | At what periods.—Weeks ending |
|---|---|---|
| 10. Ague - - | Bridgewater - | Sept. 14; Oct. 5 |
| " | Canterbury - | Sept. 14, 28 [9, 16, 30 |
| " | Chatham - | Sept. 7, 21, 28; Oct. 5, 19, 26; Nov. 2, |
| " | Bedford - - | Nov. 23, 30 |
| " | East Dereham | Sept. 14, 21 |
| " | Lincoln - - | Nov. 9, 16, 23 |
| " | Liverpool - | Sept. 14 |
| 11. Remittent Fever | Odiham - - | Oct. 12 |
| " | Bridgewater - | Sept. 7; Nov. 9, 16, 23, 30 |
| " | Saffron Walden | Every week |
| " | Thetford - - | Sept. 7, 14; Oct. 12, 26 |
| " | Pontesbury - | Nov. 30 |
| " | Alford - - | Sept. 28; Oct. 26; all Nov. |
| " | Bolton-le-moors | Sept. 14, 21, 28; Oct. 5; Nov. 2, 9, 16 |
| " | Rothbury - | Nov. 16 |
| 12. Diarrhœa - | Odiham - | All Sept.; Oct. 5; Nov. 2, 9, 30 |
| " | Bridgewater - | Sept. 14, Oct. 5, 12, 19; Nov. 9, 16, 30 |
| " | Canterbury - | Every week but Nov. 30 |
| " | Chatham - | Every week but Nov. 16 |
| " | Upper Holloway | Oct. 5 |
| " | Wanstead - | All Sept.; Oct. 5, 12 |
| " | Swansea - - | All Sept.; Oct. 5, 26; Nov. 2, 9 |
| " | Saffron Walden | All Sept.; Oct. 5, 12 |
| " | Bedford - - | Sept. 7, 14, 21; all Oct. and Nov. |
| " | Thetford - - | Sept. 14, 21; Oct. 5, 12, 26; Nov. 9, |
| " | East Dereham - | All Sept.; Oct. 5, 12, 19 [16, 23, 30 |
| " | Pontesbury - | Sept. 21, 28; Oct. 5 |
| " | Nottingham - | Nearly every week |
| " | Wrexham - | Nov. 23, 30 |
| " | Hawarden - | Sept. 7; all Oct. and Nov. |
| " | Lincoln - - | All Sept. |
| " | Alford - - | Sept. 7, 21, 28; Oct. 5, 19; Nov. 23 |
| " | Gainsborough | Every week |
| " | Liverpool - | Every week but Nov. 30 |
| " | Bolton-le-moors | Sept. 7, 14; Oct. 5, 12, 19 |
| " | West Auckland | All Sept. |
| " | Rothbury - | Sept. 14, 21, 28; Oct. 5, 12 |
| 13. Dysentery - | Odiham - - | All Sept.; Oct. 5 |
| " | Chatham - | All Sept. and Oct.; Nov. 9 |
| " | Upper Holloway | Nov. 2 |
| " | Wanstead - | Nov. 30 |
| " | Saffron Walden | Oct. 26; Nov. 2, 23 |
| " | Bedford - - | Sept. 14 |
| " | Thetford - | Sept. 7, 14, 28 |
| " | Pontesbury - | Nov. 30 |
| " | Nottingham - | Nearly every week |
| " | Lincoln - | Sept. 7, 14, 21 |
| " | Alford - - | Nov. 23 |
| " | Liverpool - | Sept. 14; Oct. 12; Nov. 9, 16, 23 |
| " | Bolton-le-moors | All Sept.; Oct. 5, 12 |
| 14. Typhus - - | Odiham - - | Nov. 30 |
| " | Bridgewater - | Nov. 23 |
| " | Canterbury - | Sept. 14, 28; Oct. 12, 26 |
| " | Chatham - | All Sept.; Oct. 12, 19, 26; Nov. 2, 16, 30 |
| " | Swansea - - | Sept. 21 |
| " | Saffron Walden | Sept. 7 [23, 30 |
| " | Bedford - - | Sept. 14, 21, 28; Oct. 5, 12, 26; Nov. 2, |

| Disease. | Where observed. | At what periods.—Weeks ending |
|---|---|---|
| 14. Typhus - - | Thetford - - | Sept. 21; Oct. 12; Nov. 16, 30 |
| „ | East Dereham | Every week |
| „ | Pontesbury - | Nov. 23, 30 |
| „ | Nottingham - | Sept. 21, 28; Oct. 5, 12, 19; Nov. 2, 9 |
| „ | Gainsborough - | Every week |
| „ | Liverpool - | Every week |
| „ | Bolton-le-moors | Sept. 14, 21, 28; Oct. 5, 12, 19 |
| „ | West Auckland | All Sept. |
| 15. Puerperal Fever | Bridgewater - | Nov. 9, 23 |
| „ | Chatham - | Nov. 30 |
| „ | Bedford - - | Nov. 23 |
| „ | Alford - - | Sept. 28; Oct. 12 |
| „ | Nottingham - | Oct. 26; Nov. 2, 30 |
| „ | Liverpool - | Sept. 14; Nov. 16 |
| 16. Carbuncle - | Odiham - - | Nov. 30 |
| „ | Bridgewater - | Sept. 28; Oct. 19; Nov. 9 |
| „ | Canterbury - | Sept. 14; Nov. 9, 16, 23 |
| „ | Chatham - | Nov. 30 |
| „ | Wanstead - | Oct. 26; Nov. 2, 9 |
| „ | Saffron Walden | Oct. 19; Nov. 16 |
| „ | Thetford - | Sept. 21; Nov. 9 |
| „ | East Dereham | Oct. 19 |
| „ | Pontesbury - | Sept. 28 |
| „ | Nottingham - | Sept. 28; Oct. 5; Nov. 16, 30 |
| „ | Wrexham - | Nov. 2, 9, 16 |
| „ | Bolton-le-moors | Sept. 28; Oct. 5; Nov. 2, 9 |

### ADDITIONAL OBSERVATIONS.

*Odiham.*—Dr. M'Intyre writes: "The quarter has been, on the whole, unusually healthy. Little rain has fallen. During the latter part of September and the beginning of October the weather was warm and muggy. Vegetables were destroyed by caterpillar to a most unusual extent. I had only one case of small-pox, very mild, after vaccination. Another case, also mild, after vaccination, occurred in a servant in the same establishment. The dysenteric cases were very severe and protracted: all occurred at the lower end of the town, where drains are emptied, and which at the time stank abominably. No death occurred in any of the cases."

*Bridgewater.*—Mr. Haviland appends the following observations:

"*Sept.* 7.—Ozone, 4. Wind N.E. Range of barometer, 0·28 in. Of thermometer, 5°; highest 62°; lowest, 57°. Very fine. Diarrhœa, neuralgia, ague, and cow-pox among cows, prevalent in the neighbourhood.

"Deaths: fever, 1; phthisis, 1; total, 2.

"— 14.—Ozone, 6. Wind N.W. Range of barometer, 0·40 in. Of thermometer, 6°; highest, 63°; lowest, 57°. Heavy rain at the end of the week.

"Deaths: disease of stomach, 1; chronic hydrocephalus, 1; phthisis, 1; total, 3.

"— 21.—Ozone, 0. Wind E. and N.W. Range of barometer, 0·22 in. Of thermometer, 1°; highest, 60°; lowest, 59°. Fine. Innumerable black flies in the air, affecting turnips, etc.: turnips stink.

"Deaths: pressure at birth, 1; natural decay, 1; phthisis, 1; hæmatemesis, 1; cancer, 1; total, 5.

"— 28.—Ozone, 17. Wind E. and S.E. Range of barometer, 0·94 in. Of thermometer, 10°; highest, 64°; lowest, 54°.

"Deaths: inflammation of the lungs, 1; phthisis, 1; total, 2.

"*Oct.* 5.—Ozone, 10. Wind. Range of barometer, 0·40 in. Of thermometer, 5°; highest, 59°; lowest, 54°. Overcast; much rain; stormy and blustering; thunder on the 4th, 11 A.M.

"Death: softening of the brain, 1; total, 1.

"— 12.—Ozone, 30. Wind N.W. Range of barometer, 0·54 in. Of thermometer, 4°; highest, 56°; lowest, 52°. Fine.

"Deaths: natural decay, 1; phthisis, 1; total, 2.

"— 19.—Ozone, 11. Wind N.W. Range of barometer, 0·60 in. Of thermometer, 9°; highest, 53°; lowest, 44°.

"Deaths: diarrhœa, 1; abscess of back, 1; dis. kidneys and lungs, 1; phthisis, 2; total, 5.

"— 26 —Ozone, 24. Wind N.W. Range of barometer, 1·13 in. Of thermometer, 6°; highest, 56°; lowest, 50°. A great deal of rain and wind all the week; a hurricane on the 26th.

"Deaths: scrofulous disease of joint, 1; pleuropneumonia, 1; convulsions, 1; total, 3.

"*Nov.* 2.—Ozone, 26. Wind N.E. Range of barometer, 0·64 in. Of thermometer, 7°; highest, 47°; lowest, 40°.

"Deaths: convulsions, 1; natural decay, 1; total, 2.

"— 9.—Ozone, 22. Wind N.W. and S.E. Range of barometer, 0·72 in. Of thermometer, 8°; highest, 48°; lowest, 40°.

"Deaths: natural decay, 1; phthisis, 1; total, 2.

"— 16.—Ozone, 0. Wind S.E. and E. Range of barometer, 0·41 in. Of thermometer, 7°; highest, 46°: lowest, 39°.

"Deaths: phthisis, 3; fever, 2; mesenteric disease, 1; unknown, 3; croup, 1; disease of stomach, 1; asthma, 1; convulsions, 1; total, 13.

"— 23.*—Ozone, 0. Wind N.E. Range of barometer, 0·42 in. Of thermometer, 5°; highest, 40°; lowest, 35°.

"Deaths: disease of heart, 1; fever, 3; pneumonia, 2; hydrocephalus, 1; total, 7.

"— 30.†—Ozone, 2. Wind N.E. Range of barometer, 0·23 in. Of thermometer, 5°; highest, 41°; lowest, 36°.

---

* It is remarkable, that during the last three weeks of November, when scarcely any ozone was registered, the mortality increased to a great extent.

† In a note from J. C. Parker, Esq., I am informed that "there has been a great number of cases of remittent and continuous fever of a low type, ***on one side only*** of the north side of West Street, Bridgwater. These cases followed the opening of an old drain on all that side of the street, for the purpose of making it deeper and wider." Probably when the laws which regulate the presence of ozone in the atmosphere are better known, we shall be able to direct the public as to the time of opening drains, cesspools, etc. Even now I would suggest that no attempt should be made during *calm* weather, but that advantage should be taken of boisterous, windy weather, especially when it blows from south, south-west, west, or north-west, which winds my experience leads me to believe the most *ozoniferous*.

"Deaths: phthisis, 1; pneumonia, 1; convulsions, 1; natural decay, 1; hernia, 1; hydrocephalus, 1; total, 6.

*Canterbury.*—Mr. Rigden writes: "I have not had cases of scarlet fever under my own care since June; but cases of this disease are reported to have been among the military in barracks; and three deaths from this disease have been registered as occurring there during the past month. Measles, after being absent from the city for about two years, has suddenly shown itself again since the middle of November. Small-pox has continued to prevail since the last quarter's report; the more severe cases being in three persons unvaccinated. Hooping-cough has been prevalent during the last quarter, and in a few cases has terminated fatally by pneumonia supervening, or by producing exhaustion. Several cases of erysipelas have been brought under treatment. Diarrhœa has been prevalent, but not of a choleraic character. Of typhus fever and carbuncle several cases have occurred."

*Chatham.*—Dr. F. J. Brown says: "During the last quarter there were two exceedingly severe cases of typhoid fever. The adynamia was extreme, and purpura appeared in one of the cases. One boy, aged nine years, died on the twenty-sixth day. He had epistaxis, and was seized with typhoid fever in the week ending 12th of October: one lad, aged sixteen years, was seized in the week ending 19th October. The fever turned on the fourteenth day, and he recovered. He had purpura. During his convalescence he had pyelitis.

"Eight cases of serous cholera occurred during the quarter. Three were fatal. The fatal cases were furnished by one infant, one child, and one old woman that nursed a man that died of cholera, in November. The old woman appeared to communicate the disease to two women residing in a house in a different part of the town, to which house she had been removed previously to her death. No other cases occurred about this period.

"Three cases of erythema nodosum were observed in November. The disease affected girls, and was marked by curious symptoms. One patient had congestion of the lungs and pains in the abdomen. Another girl had congestion of the kidneys, on the subsidence of the skin affection.

"One case of metria occurred in the last week of the quarter. The woman got out of bed to prevent a drunken quarrel in the room. She was seized with rigors, and her pulse became rapid. The abdomen was slightly painful, but not swelled. Death took place in fifty-eight hours.

"Two cases of phlebitis of the leg—one of them being accompanied by white swelling—were observed in the week ending 16th of November.

"A case of small-pox, attended by purpura, occurred in the week ending 26th of October. There was no blood in the pocks themselves. The child recovered. I did not see the case.

"Congestion of the head was frequently met with in October.

There were many cases, especially in one night; namely, the night of the 21st, and early morning of the 22nd.

"The amount of haze during the month of November was great; namely, on fourteen days."

*Saffron Walden.*—Mr. Spurgin writes: "Public health, on the whole, during the quarter, has been good, with the exception of the prevalence of scarlet fever, which has been fatal in some families, but not from want of ventilation or care on the part of attendants. The fogs and damp of November, with the want of sunshine, have caused the prevalence of colds and bronchial affections during the latter part of the quarter. Vegetation has been unusually protracted, and disease has been more partial among the esculent roots, particularly potatoes; turnips and mangel wurzel being unusually fine, and large."

*Swansea.*—Mr. Michael writes: "This quarter was extremely healthy. Scarlatina of the mildest character occurred, often only known by its sequelæ, and very slight. One case of suppressed scarlet fever proved fatal in thirty-six hours. Three cases of continued fever occurred. There was one interesting case of small-pox, consentaneous with pustular syphilitic eruption on the face: the small-pox pustules left pits. One other imported case of confluent small-pox proved fatal."

*Bedford.*—Dr. Barker writes that "Scarlet fever, of an unusually malignant and fatal character, has prevailed during the entire quarter, in Bedford and some of the neighbouring villages. Although no week has passed without fresh cases of the disease, it is evidently decreasing in number during the last two or three weeks. Other observations on this epidemic will be reserved for a more extended report. Cases of catarrh and influenza have been frequent since the damp and cold weather commenced, with the wind principally N.E. Typhus and typhoid fevers have been fatal in several cases. I have noticed during the last four weeks an unusual number of apoplectic and paralytic seizures in my practice."

*Thetford.*—Mr. Bailey makes the following observations: "Those diseases which are considered specifically contagious, have during this quarter been very epidemic. Scarlatina in the last seven weeks has been universally prevalent, invading most families, mild in its progress, and, except in a few cases, unaccompanied by anasarcous effusions; no fatal case has occurred. Rubeola has become also very prevalent throughout the quarter, especially in the town. In adults, it has been very severe, the inflammatory action high, with most severe cough, requiring most active treatment. With children, the disease has been of a milder character. Infants have not escaped, and two cases terminated fatally. During the four months, pertussis has been under medical treatment, in many cases following the measles. With infants, it has proved fatal from suffocation. For many years I have not observed the mumps so generally attacking adults as well as younger persons. In the last month, new cases came daily under observation. The simultaneous occurrence of these specifically contagious diseases is somewhat singular.

Referring to my meteorological registers of some years past, I find that during the prevalence of the north and north-east winds in the autumn, with the dew-point high, the air being saturated with moisture above the average, scarlatina and measles were very prevalent, the former running into scarlatina anginosa, which carried off many children, sometimes two or three in a family. The measles were at that time very severe and fatal. Such was the epidemic constitution of the season. Had the autumn been dry and cold, probably we should have escaped these diseases.

"From the damp state of the atmosphere, together with some cold and frosty nights, influenza and catarrhs have been produced. In elderly people, much prostration has been occasioned by the severe symptoms. Diarrhœa has also been extremely prevalent, evidently arising from atmospheric causes: as well as cynanche tonsillaris, rheumatism, and bronchitis in children and elderly persons. Several cases of remittent fever and typhus have come under treatment in the labouring classes, who are incapable of resisting the atmospheric changes. These have been isolated, not arising from contagious causes. A fatal case of phthisis pulmonum, and one case of apoplexy, have occurred during the last month; the latter in a constitution predisposed to the disease from hereditary constitution, being the third daughter so taken off. Other inflammatory diseases have occurred during the quarter, not enumerated in the schedule; viz: pleuritis, hepatitis, ascites, hæmoptysis, convulsions from dentition, hæmorrhagica purpurea, syphilis, tic douloureux, colonitis, and hydrocephalus acutus. These do not arise from epidemic causes, but may be considered of a casual nature.

"During the last month, much sickness has prevailed with the cattle. Horses have been attacked with inflammation of the lungs and bowels; the strangles have attacked many horses, and the murrain amongst sheep and lambs has been very prevalent, proving very fatal in different flocks in this neighbourhood; one flock losing five or six score in a few days. From the severe frosty nights, the foliage has suddenly dropped from the trees, and we have early had the wintry aspect around us."

"Registrar's report of the fatal diseases of the quarter:

| *September.* | | *October.* | | *November.* | |
|---|---|---|---|---|---|
| Enteritis | 1 | Atrophy | 3 | Dropsy | 1 |
| Decay of nature | 1 | Diarrhœa | 1 | Atrophy | 2 |
| Phlebitis | 1 | Epilepsy | 1 | Pertussis | 2 |
| Phthisis pulmonum | 1 | Pneumonia | 1 | Hydroceph. acutus | 1 |
| Cynanche maligna | 1 | Decay of nature | 1 | Synochus | 1 |
| Suffocation | 1 | Phthisis pulmonum | 1 | Pleuritis | 1 |
| Paralysis | 1 | Enteritis | 1 | Cerebritis | 1 |
| Convulsions | 1 | Debility | 1 | Apoplexy | 1 |
| Debility in new-born infant | 1 | | | Decay of nature | 2 |
| | | | | Debility | 2 |
| | | | | Measles | 2 |
| | 9 | | 10 | | 16 |

*Pontesbury.*—Mr. Eddowes writes: "Boils have been very common in this neighbourhood for the last two years. Typhus has attacked one family in a neighbouring parish: one of the children caught it in Shrewsbury about the beginning of October, and took it home. His father caught it, and was ill a month: two of his other children also caught it; the only remaining one had diarrhœa the week before last, but went about, and is now convalescent, but weak. The mother is as yet free The man became a pauper during his illness, and was attended by a neighbouring practitioner, who is now unwell; so last week and this I visited them for him. He has no other case of typhus in his practice."

*Nottingham.*—Dr. Robertson writes: "Like the whole year, the quarter just concluded has been comparatively healthy. The principal diseases were catarrh, influenza, diarrhœa, and dysentery; the last especially in the lower quarters of the town. None of these, however, though pretty general, have prevailed to a great extent. The two cases of puerperal fever which occurred during the quarter could not be traced to any peculiar exciting cause.

"The *notabilia* in plants were only that, in September, the potato disease showed itself, and the mildew appeared extensively on vines; and in October an immense quantity of caterpillars were noticed, especially on the cabbage tribe.

"Cattle have been unusually healthy. Beyond the ordinary cases of lameness, etc., a few cases of common catarrh, and during the last fortnight some cases of pleuropneumonia, have been the only diseases requiring active treatment.

"I subjoin a table combining the meteorological and mortality observation for the quarter.

| | Sept. | Oct. | Nov. |
|---|---|---|---|
| Mean temperature | 56·2 | 48·6 | 49·1 |
| Mean pressure (corrected to 181 ft.) | 29·970 | 29·465 | 29·867 |
| Amount of rain | 0·6 | 4·5 | 1·0 |
| Amount of cloud | 5·7 | 7·0 | 8·2 |
| Direction of the wind | W to N | Var. | Nthly. |
| Amount of ozone (Schonbein's scale) | 3·3 | 3·1 | 3·1 |
| *Number of diseases | 338 | 327 | 498 |
| *Number of deaths | 118 | 106 | 105 |

"The table includes such new cases of disease as may be traced to *originate* on a certain day. The record is compiled from returns, kindly furnished to me by several medical men residing in the town, for weekly publication in connexion with meteorological observations in the *Association Medical Journal.*"

*Wrexham.*—Dr. E. Williams states: "Four cases of whitlow came under my observation during November. Slight punctures and cuts seemed more disposed to suppurate than to heal, about the same time. Boils were also prevalent.

"The potato disease made its appearance earlier this year, attacking what we commonly call in this neighbourhood the 'second early' crop, which escaped in previous years. The winter potato,

* Taken, by the kind permission of the Sanitary Committee, from the reports of the district registrars.

which used to suffer most, has presented very little of the disease this year."

*Lincoln.*—Mr. Lowe writes: "Hooping-cough has been very prevalent during the quarter, in children under twelve months, frequently resulting in pneumonia and death. We have had a few cases of malignant scarlet fever. In September, dysentery in children was very common, and frequently fatal. We also had many cases of simple eruption and furunculi; and whole families of children were affected with ulcers of the gums, fauces, and lips. During the damp weather of November we had several cases of ague, in patients who had been previously affected with it some years ago."

*Alford, Lincolnshire.*—Mr. West states: "There has been very little sickness in my district during the past quarter—at least, of the epidemic kind. I have had several cases of remittent fever, which at one time threatened to prevail very extensively. At present, I think, we have less of it."

*Gainsborough.*—Dr. Mackinder makes the following observations: "Though varioloid, hooping cough, rheumatism, and typhoid fever have existed throughout the quarter, and have interrupted the happiness of a few families, we have had nothing like an epidemic. The extreme poor have enjoyed an immunity from infectious disease; and notwithstanding the high war-price of provisions, and scarcity of work, the tenants of our narrow court-yards would not suffer by a comparison with the inhabitants of localities reputed healthy. Mr. Sharp informs me that there have been twenty-four cases of fever entered on the books of the Dispensary, none of which proved fatal.

"Mr. Dyson has furnished the subjoined meteorological report: 'The Meteorological Report for the quarter ending 30th November, does not present any remarkable vicissitude. During the month of September the sky was generally free from cloud, and less than half an inch of rain fell in the entire month, and fog was prevalent only on three days. October was rainy, and the atmophere filled with moisture, lunar halos being frequently visible: the amount of rain was nearly $4\frac{1}{2}$ inches in depth, and the nights frequently foggy. The atmosphere was, for the most part, clear and calm during November, with occasional fogs; rain fell to little more than the depth of an inch, making six inches in the quarter. The highest temperature in September was 73°, the lowest 38°; the highest in October 65°, and lowest 32°; in November the highest was 49°, and the lowest on the night of the 15th, 25°, the range in each month being considerable. As the atmosphere was generally calm, very little ozone was developed throughout the quarter; except on the last three days in October, and the first two in November, when the weather was boisterous and ozone was developed in excess, the test-paper being entirely discolored several times in the twenty-four hours, especially on the 31st of October."

*Liverpool.*—Mr. Bickerton has made the following remarks:

"*Sept.* 7.—The mortality of the borough has been less in this than during the three previous weeks. The deaths from all causes during the week having been 229, being 20 below the ordinary average of corresponding weeks in former years. Of the 229 deaths, 28 occurred from scarlatina, 46 from diarrhœa, 13 from typhus, 6 from measles, and 1 from small-pox, the patient not having been previously vaccinated. There were 40 deaths from disease of the lungs.

"— 14.—During this week there were 223 deaths from all causes; 95 being from zymotic diseases.

"— 21.—During this week the number of deaths was 203. From scarlatina there were 30; from diarrhœa, 27; from rubeola, 12; from typhus, 6; from syphilis, 2; and from diseases of the lungs, 29.

"— 28.—The number of deaths for this week was 234; from scarlatina, 31; from diarrhœa, 30; from rubeola, 8; from typhus, 4; from syphilis, 2; from variola, 1 (previously vaccinated); and from diseases of the lungs, 51.

"During the quarter ending September 29, 1855, 2,874 deaths were registered in the borough, including 208 sudden and violent deaths, and deaths in hospitals. This number is 530 less than the average mortality of former years, excluding epidemic cholera.

"*Oct.* 6.—In this week the number of deaths was 200, (average of corresponding week of previous years, 241). From scarlatina there were 27; from diarrhœa, 30; from rubeola, 8; from typhus, 5. 131 deaths occurred below the age of five years.

"— 13.—In this week the number of deaths was 192, (average of former years for corresponding weeks, 222). From scarlatina there were 41; from diarrhœa, 14; from rubeola, 7; from typhus, 19; from variola, 2; diseases of lungs caused 37 deaths.

"— 20.—In this week the number of deaths registered, was 243; the average of former years being 213. There occurred from scarlatina, 42 (20 in Toxteth Park); from typhus, 10; from diarrhœa, 12; from rubeola, 9; from pertussis, 10; from syphilis, 4; from small-pox, 1, (aged eighteen, had been vaccinated in infancy). There were 45 deaths from diseases of the lungs; 153 out of the 243 deaths occurred in persons below the age of five years. The weather had changed from fine to much rain and wind; and the temperature had been low.

"*Nov.* 22.—During the four weeks ending November 22, 1855, the deaths from all causes were respectively, 229, 205, 210, 238. Allowing for increase of population, the average was below that for the same period in previous years. The deaths from zymotic diseases, in the four weeks were from scarlatina, 360; from diarrhœa, 160; from rubeola, 42; from pertussis, 42; from typhus, 38; from croup, 35; from variola, 13; from varicella, 4; from mumps, 1; from glanders, 1. The deaths from hooping cough in the last week were more than in any week since last January. Diseases of the lungs caused 220 deaths in the four weeks, of which 105 were from phthisis pulmonalis.

"— 27.—In this week the number of deaths from all causes was 245. Scarlatina was diminishing in Toxteth Park, which had been fixed for months. From diseases of the zymotic class there were 49 deaths during the week. One adult and six infants died from syphilis, being a greater number than any recorded in any previous week. Sixty-five deaths occurred from diseases of the lungs, more than in any week during the preceding five months.

"The temperature was below the average. No rain, wind E.N.E."

*West Auckland.*—Mr. Todd writes: "Scarlatina was prevalent during the whole of October and November; generally of a mild character, but in some instances complicated with bronchitis and ascites. One case occurred to a man aged 40, who cannot recollect ever having had the disease before. This case was attended by a copious cutaneous eruption, high fever, and slight affection of the throat. One case of very severe phlegmonous erysipelas of the leg occurred in September, which ultimately did well. Diarrhœa was prevalent during September, but disappeared in October, when the weather became more dry, cold, and frosty. Continued fever of a mild character has been prevalent for some time, and declined about the beginning of October. In most of the cases there was more or less of gastro-enteric affection. The origin of the fever was paludal: there was no contagion.

*Rothbury.*—Mr. Summers writes: "The past quarter has been, in this district, remarkably healthy. Diarrhœa, which usually prevails in the autumn to a considerable extent, can scarcely be said to have been epidemic here this year; the cases which did occur in the weeks noted, being very few and very mild. It is worthy of remark that most fruits have been remarkably *plentiful* this season; the chief exception to this being in the shape of plums, and stone-fruit generally, which have been scarce in this part of the country. I have only noted one case of remittent fever; and that occurred in a child aged about ten years, who had just come from Newcastle, where her mother was lying ill of 'bilious fever.' The weather has been on the whole seasonable, and farm stock has been quite exempt from epidemic diseases. Scarlet fever prevailed during September and October, with some severity, in the neighbouring town of Morpeth, and generally to the eastward of this place; but I heard of no cases within four or five miles of Rothbury. The deaths in the parish of Rothbury, comprising a circle of nearly five miles on all sides around the village, and a population in 1851 of 2,545, have been six in the quarter just ended. Five cases were adults, who died of chronic diseases; the remaining one was an infant, who died from acute hydrocephalus."

---

